# Supplementary material for: Complexome profiling of the Chlamydomonas psb28 mutant reveals TEF5 as an early PSII assembly factor
Source: Plant Cell. 2025 Mar 17;37(6):koaf055. doi: 10.1093/plcell/koaf055 (PMC12164590; doi:10.1093/plcell/koaf055)
Supplement: koaf055_Supplementary_Data [file koaf055_supplementary_data.zip › psb28_interactive.zip!/plots/intensity/qualityPlots/peptides/SFA1_Cre07.g332950.t1.2.html]

### Cre07.g332950.t1.2

Trivial name: SFA1  
SeqCoverage [median]: 9.80  
Mass: 31.18 kDa  
  
 

| Sequence | MissedCleavage | Mass [Da] | wtIntensity | muIntensity | Score |
| --- | --- | --- | --- | --- | --- |
| AALALEREER | 1 | 1156.62 | 0.00 | 584.35 | 56.26 |
| ALQEGLK | 0 | 757.43 | 548.98 | 447.35 | 92.69 |
|  |  |  | 549.0 | 1031.7 |  |
